# Metformin exhibits the anti-proliferation and anti-invasion effects in hepatocellular carcinoma cells after insufficient radiofrequency ablation

**DOI:** 10.1186/s12935-017-0418-6

**Published:** 2017-04-24

**Authors:** Qingyun Zhang, Jian Kong, Shuying Dong, Wenlei Xu, Wenbing Sun

**Affiliations:** 1 Department of Hepatobiliary Surgery, Beijing Chao-yang Hospital, Capital Medical University, Beijing, 100043 China; 2 Department of General Surgery, Affiliated Hospital of Chengde Medical University, Hebei, 067000 China

**Keywords:** Hepatocellular carcinoma, Insufficient radiofrequency ablation, Metformin

## Abstract

**Background:**

The mechanisms and prevention of progression of hepatocellular carcinoma (HCC) after insufficient radiofrequency ablation (RFA) has been preliminarily investigated, therefore, new strategy needs to be investigated to prevent the process. Whether metformin could be used to inhibit the growth of HCC after insufficient RFA and further prevent the progression of residual HCC remains unclearly.

**Methods:**

MTT assay, colony formation assay and transwell assay were used to observe the cell viability, migration and invasion. Western blot and immunohistochemistry methods were used to observe the expression of proteins. Xenograft model was used to evaluate the growth of HCC cells in vivo.

**Results:**

Metformin inhibited the enhanced proliferation, migration and invasion of HepG2 and SMMC7721 cells after insufficient RFA (named as HepG2-H and SMMC7721-H). Metformin deregulated the expression of p-Akt in HepG2 and SMMC7721 cells after insufficient RFA through AMPK/PTEN pathway. HepG2-H cells also exhibited larger tumor size in vivo. Higher expression of Ki-67 and CD31 and lower expression of E-cadherin were observed in HepG2-H tumors. Metformin blocked the enhanced growth of HepG2 cells in vivo after insufficient RFA. Metformin had no apparent toxicity on nude mice.

**Conclusions:**

Metfromin inhibited the growth of HCC cells after insufficient RFA, and may be used to prevent the progression of HCC after RFA.

**Electronic supplementary material:**

The online version of this article (doi:10.1186/s12935-017-0418-6) contains supplementary material, which is available to authorized users.

## Background

Hepatocellular carcinoma (HCC) is one of the most common malignancies worldwide with rising incidence in both Eastern and Western countries [1]. The treatment modalities for HCC include surgical resection, radiofrequency ablation (RFA), and liver transplantation [2]. RFA was shown to achieve comparable overall survival and better tolerability for early HCC, and is recommended as the priority treatment for very early-stage HCC with impaired liver functional reserve [3]. However, suboptimal RFA treatment for HCC has been reported as a risk factor of early diffuse recurrence [4].

Several researches have explored the mechanisms and prevention of progression of HCC after insufficient RFA [5, 6]. Our previous study demonstrated that sorafenib suppressed the epithelial-mesenchymal transition of HCC after insufficient RFA. Nevertheless, sorafenib is the first and only molecular targeted therapy approved for use in advanced HCC by the U.S. Food and Drug Administration, which also causes multiple human toxicities, including use-limiting anorexia, GI bleeds and hand–foot syndrome [7]. Consequently, new strategy needs to be investigated to prevent the process.

Metformin is recommended as first-line therapy for all newly diagnosed Type 2 Diabetes Mellitus (T2DM) patients. Some epidemiologic data have highlighted the positive effects of metformin to reduce cancer incidence and mortality [8–11]. Anticancer effects of metformin can be divided into two non-exclusive categories: an indirect effect by reducing the blood glucose and insulin levels, and a direct effect on cancer cells, partially through the activation of AMPK [12]. Metformin also showed promising prospect in the treatment of HCC. Metformin conferred risk reduction for developing HCC recurrence after resection [13]. Metformin also inhibited cell proliferation, invasion, angiogenesis, and induced apoptosis in HCC [14–17]. Metformin also enhanced the effect of sorafenib, arsenic trioxide and 5-fluorouracil, and reversed multidrug resistance of HCC [18–22]. Whether metformin could be used to inhibit the growth of HCC after insufficient RFA and further prevent the progression of residual HCC remains unclearly.

In the present study, we observed the effects of metformin on cell proliferation, migration and invasion of HCC cell lines (HepG2 and SMMC7721) after insufficient RFA in vitro. Furthermore, we analyzed the influences of metformin on changes of PCNA, E-cadherin, VEGF, and Akt signaling pathways involved in the process in HCC cells after insufficient RFA. We also performed in vivo experiments to study the effect of metformin on the growth of HCC cells after insufficient RFA in a BALB/c nu/nu mice model.

## Methods

### Cell line and cell culture

The human HCC cell line (HepG2 and SMMC7721) was obtained from the American Type Culture Collection (ATCC; Manassas, VA, USA). Cells were cultured in high-glucose Dulbecco’s modified Eagle medium (DMEM) supplement with 10% fetal bovine serum (FBS), 100 U/ml penicillin and 100 μg/ml streptomycin in humidified atmosphere of 5% CO_2_ at 37 °C.

### Reagents and antibodies

Metformin was obtained from Sigma-Aldrich (St. Louis, MO, USA). Horseradish peroxidase (HRP)-labeled anti-mouse and anti-rabbit secondary antibodies were from Santa Cruz (Dallas, TX, USA). Phospho-anti-Akt, PTEN and phospho-anti-AMPK antibodies were purchased form Cell signaling (Beverly, CA, USA). Anti-Ecadherin, VEGF, PCNA, t-Akt, t-AMPK and β-actin were bought form Abcam (Cambridge, TX, USA).

### Insufficient RFA in vitro

Insufficient RFA was simulated in vitro as described before [23]. Simply, HCC cells were seeded into the 6-well plates (5 × 10^4^ cells/well). After 24 h, the plates were sealed and submerged in a water bath set to 47 °C for 5 min. Thereafter, cells were allowed to recover, and when the surviving populations reached 80% confluence, cells were propagated into the 6-well plates and exposed to above treatment for 10 min. Then the process was repeated and cells were sequentially exposed to above treatment for 15, 20 and 25 min. Cells surviving from the treatment were designated as HepG2-H and SMMC7721-H cells.

### MTT

Hepatocellular carcinoma cells were trypsinized and seeded into 96-well plates at a density of approximately 3000 cells per well. Twenty-four hours later, adherent cells were treated metfromin. After 72 h incubation, MTT reagent was added to the cells (0.5 mg/ml), and cells were then incubated for 4 h at 37 °C. The cells media were removed and 150 μl DMSO were added to each well followed by gentle shaking of the plates to dissolve the formazan crystals. The optical density (OD) was then measured using an automated ELISA plate reader at 570 nm.

### Colony formation assay

Briefly, HepG2, HepG2-H, SMMC7721 and SMMC7721-H cells were recultured in 6-well plates (1000 cell per well), incubated with or without metformin for 24 h, and continuously grew in complete medium for 2 weeks. Then, the cell colonies were washed with PBS, fixed by methanol, and stained with crystal violet (Beyotime, Nantong, China). The colonies were counted and compared with untreated cells.

### Transwell assay

Cell migration assays were operated by a modified Boyden chamber (Costar-Corning, New York, USA). HCC cells (5000 cells per well) were added into the upper chamber, and 500 μl DMEM with 10% FBS were added into the lower chamber. After 30 min incubation, metformin were added to the upper chamber. The chambers were incubated under the usual culture conditions for 24 h. After removing the filter inserts and the cells on the upper side of the filter, the migrated cells on the lower chamber were fixed with methanol, stained with crystal violet for 30 min, washed with PBS, and captured using photographed under an inverted fluorescence microscope (Olympus IX51) equipped with an Olympus Qcolor 3 digital camera (Olympus). Migration was assessed by counting the number of stained cells from 10 random fields at 200× magnification.

For cell invasion assay by transwell assay, each insert needed was precoated with Matrigel. The others steps were similar to cell migration assay.

### Western blot

Cells were collected and cell lysis was performed by using RIPA lysis buffer including proteased inhibitors on ice. The extracted protein was quantified by bicinchoninic acid quantification assay. Then, the total cellular proteins were subjected to SDS-PAGE gel and transferred to nitrocellulose membranes. The membranes were blocked with 5% non-fat milk for 2 h and then incubated with respective primary antibody overnight at 4 °C. Following washing three times with TBS-T for 10 min, the membranes were incubated with the appropriate HRP-conjugated secondary antibody for 1.5 h at room temperature. The bands were captured with SuperSignal West Pico substrate (Thermo scientific, Rockford, IL, USA).

### Xenograft model

Pathogen-free male BALB/c nu/nu mice (4–6 weeks of age) were obtained from Vital River Laboratories (Beijing, China). HepG2 and HepG2-H cells (5 × 10^6^) were suspended in 200 μl serum-free DMEM and matrigel (1:1) and then injected subcutaneously into the upper right flank region of 20 nude mice. After 1 w, mice were treated with metformin by oral route (200 mg/kg/day), or PBS as control every day for up to the 24th day. Tumor size was evaluated with calipers every 3 days. Mice were euthanized, and tissues were removed for fixation in the 4% paraformaldehyde for histologic examination and immunohistochemical staining.

### Immunocytochemistry

Tumor specimens were immediately removed from sacrificed mice and prepared for immunohistological examination. Tumors were fixed in 4% paraformaldehyde overnight, embedded in paraffin and sectioned to a 6 μm sections thickness. Tumor sections were deparaffinized via immersion in xylene, dehydrated in a graded series of ethanol, and washed with distilled water. Thereafter, tumor sections were boiled in at 92 °C in EDTA (10 mmol/l, pH 8.0) for 10 min and cooled at room temperature. To inhibit endogenous peroxidase activity, tumor sections were incubated with 0.3% hydrogen peroxide for 12 min. Tumor sections were then blocked with 5% goat serum for 60 min at 37 °C, and then incubated overnight with primary antibodies at 4 °C. Tumor sections were probed with peroxidase-conjugated secondary antibodies and incubated with DAB until the desired stain intensity developed. Finally, the slides were counterstained with hematoxylin and mounted. The slides were examined with Nikon Eclipse Ti microscope under a 200× objective.

### Statistical analysis

All values are expressed as the mean ± SEM. The data were analyzed using Student’s t test or the ANOVA test. A P value of <0.05 was considered statistically significant. GraphPad Prism (GraphPad Software Inc., San Diego, California, USA) was used for these analyses.

## Results

### Metformin suppressed the insufficient RFA-induced proliferation, migration and invasion of HCC cells in vitro

**Fig. 1:**
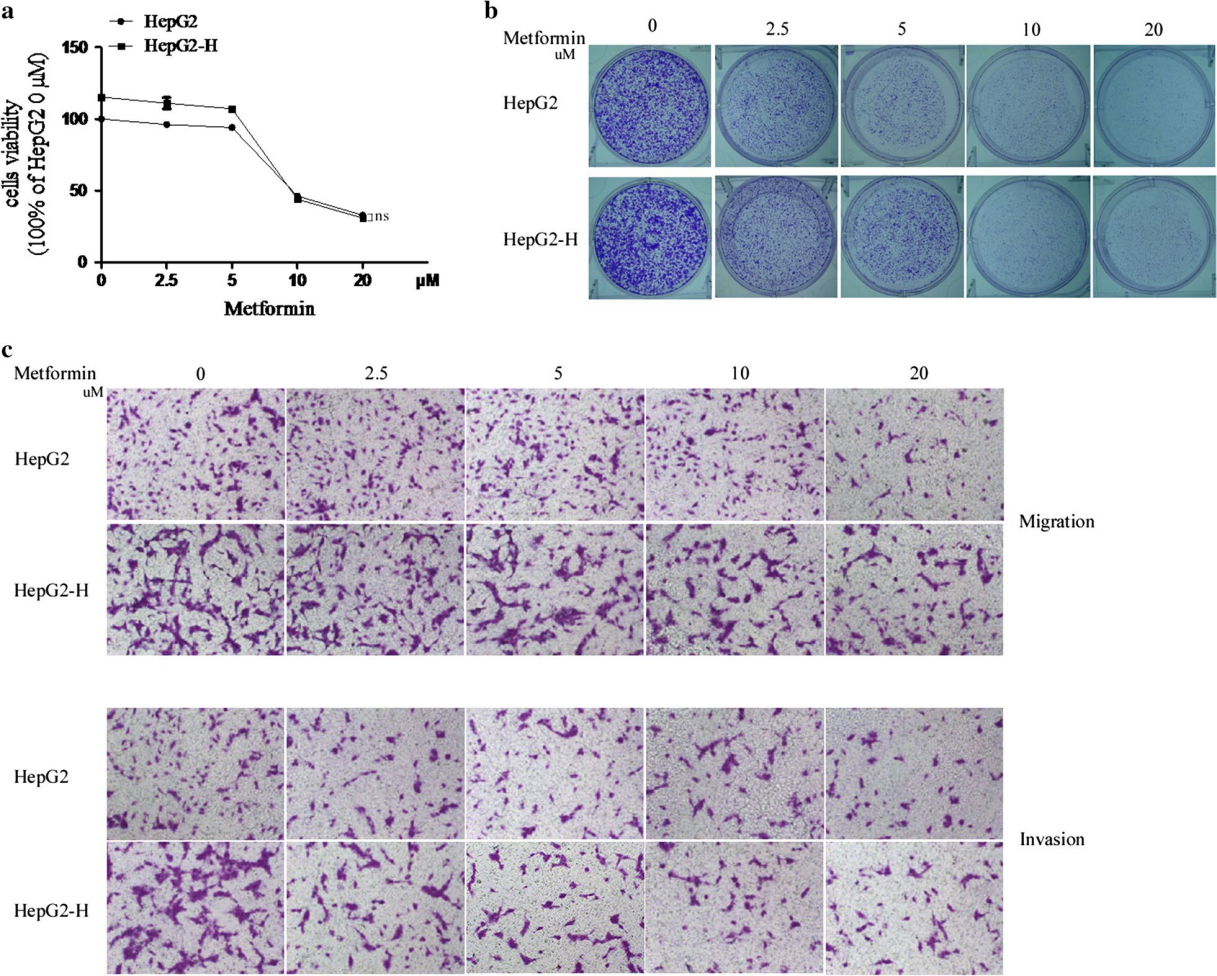
Metformin suppressed the insufficient RFA-induced proliferation, migration and invasion of HepG2 cells. HepG2 cells were treated with insufficient RFA (47 °C 5, 10, 15, 20 and 25 min) gradually. Residual HepG2 (named as HepG2-H) cells were collected and used for the next experiments. **a** The effect of metformin on proliferation rate of HepG2 and HepG2-H cells was evaluated by MTT assay. *Error bars* represent the SEM of data obtained in five independent experiments. **b** Colony formation ability of HepG2 and HepG2-H cells after the treatment of metformin was assessed. **c** The effect of metfromin on migration and invasion of HepG2 and HepG2-H cells were shown. *Error bars* represent the SEM of data obtained in three independent experiments. *ns* no significance

Our previous study demonstrated that insufficient RFA promoted proliferation, migration and invasion of HepG2 cells, therefore, we next explored whether metformin could abrogate the process. As the same in our previous study, HepG2 cells after insufficient RFA in vitro (named as HepG2-H) showed higher proliferation rate compared with HepG2 cells at 72 h (Fig. 1a). Metformin impeded the proliferation rate of HepG2 and HepG2-H cells in a dose-dependent manner (Fig. 1a). Metformin (5, 10 and 20 μM) abrogated the distinction of proliferation rate between HepG2 and HepG2-H cells (Fig. 1a). A colony formation assay was done to test whether metformin affected clonogenic potential, which is an important characteristic of tumor growth in vivo. As shown, more colony numbers were found in HepG2-H cells compared with HepG2 cell and metformin reduced the number of colonies in a dose-dependent manner (Fig. 1b). Furthermore, insufficient RFA induced an increase number of cell migration and invasion in HepG2 cells, which was also repressed by metformin in vitro (Fig. 1c).

Similar results were also found in SMMC7721 cells (Additional file 1: Figure S1).

### Metformin may regulate HCC cells proliferation, migration and invasion after insufficient RFA by promoting AMPK/PTEN/Akt pathway

**Fig. 2:**
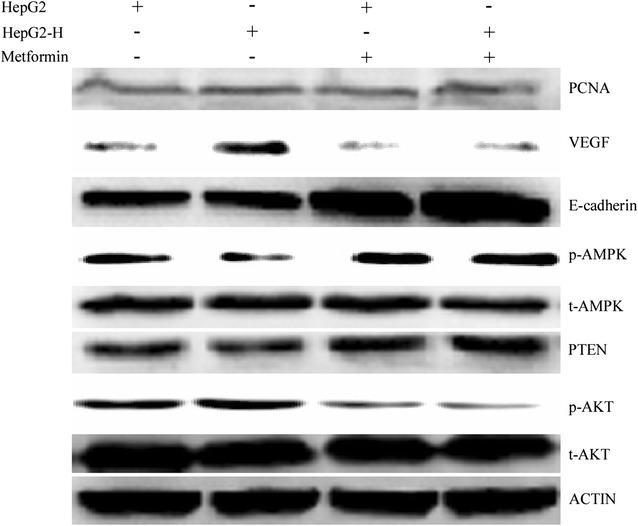
Metformin may regulate HCC cells proliferation, migration and invasion after insufficient RFA by promoting AMPK/PTEN/Akt pathway. Metformin was used to treat HepG2 cells, and western blot was used to determined the expression of p-AMPK, PTEN, p-Akt, E-cadherin, PCNA and VEGF

Up-regulation of VEGF and PCNA and down-regulation of E-cadherin could be observed in HepG2-H cells at protein levels (Fig. 2). Treatment of cells with metformin increased E-cadherin expression, and decreased the PCNA and VEGF expression (Fig. 2). To explore the possible mechanism of metformin involved in the process of HCC cells after insufficient RFA, the AMPK/PTEN/Akt pathway was tested. Significantly increased expression of p-Akt and decreased expression of p-AMPK and PTEN were found in HepG2-H cells compared with HepG2 cells (Fig. 2). Furthermore, metformin decreased the expression of p-Akt and increased the expression of p-AMPK and PTEN in HepG2 and HepG2-H cells (Fig. 2).

Similar results were also found in SMMC7721 cells (Additional file 2: Figure S2).

### Metformin ameliorated the process that insufficient RFA enhanced the growth of HCC cells in vivo

**Fig. 3:**
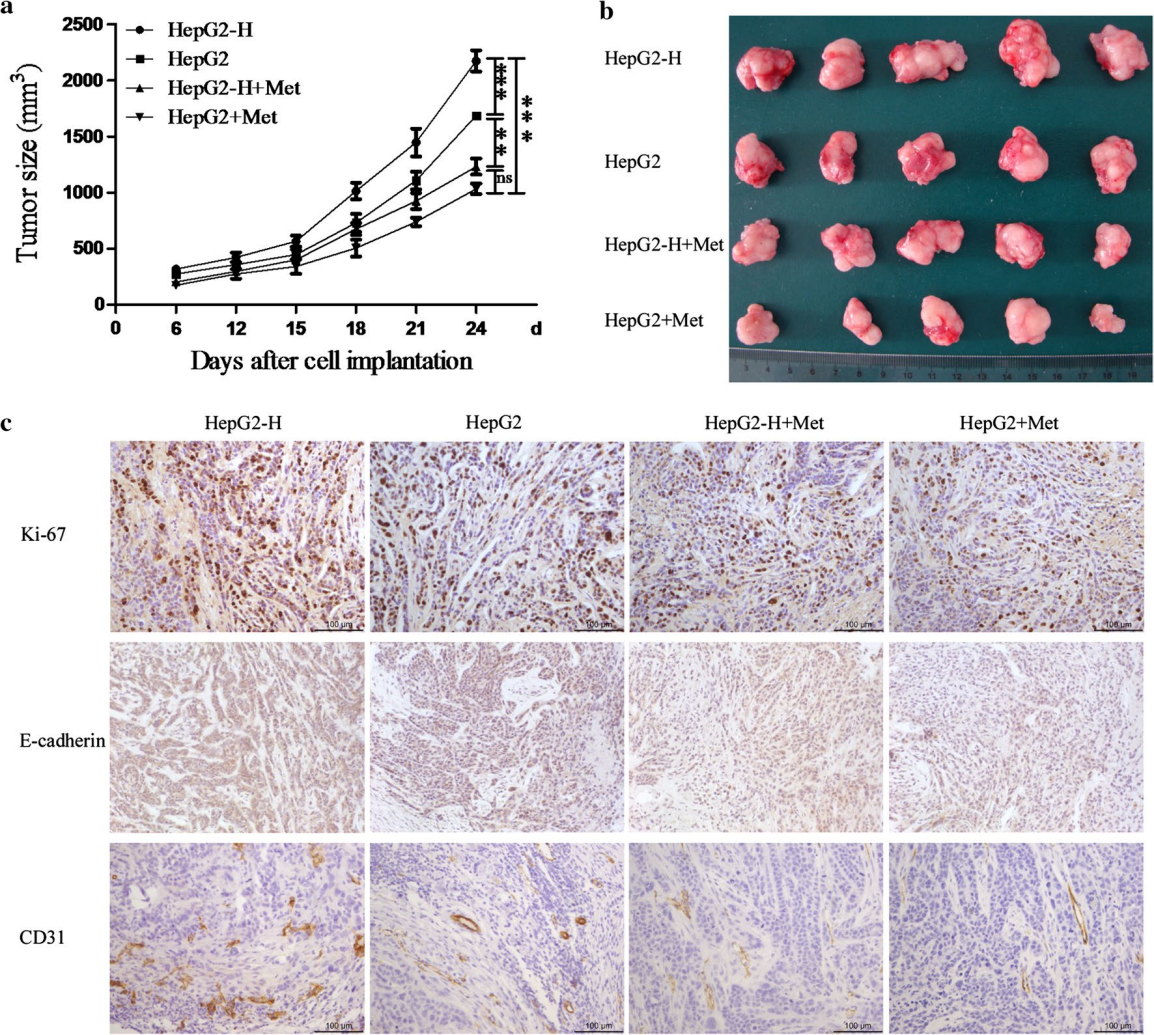
Metformin ameliorated the process that insufficient RFA enhanced the growth of HCC cells in vivo. HepG2 and HepG2-H cells were injected subcutaneously into the *upper right* flank region of nude mice, treated with or without metformin, and tumor volume was measured. **a** Tumor volume was measured with a caliper rule every 3 days. Data were presented as the mean tumor volumes of mice. **b** Tumor size of the 24th day after cell implantation was displayed. **c** Tumor sections were stained for Ki-67, E-cadherin, and CD31. Representative images of the immunohistochemistry assay were shown (200×)

In HepG2-H group, tumors grew more rapidly compared with HepG2 group, and metformin significantly inhibited the growth of HepG2 and HepG2-H tumors (Fig. 3a, b). Metformin ameliorated the difference of tumor growth between HepG2 and HepG2-H cells (Fig. 3a). Significant increases of cell proliferation were observed in HepG2-H cells, and metfromin inhibited the process (Fig. 3c). Tumor microvessel density was analyzed using CD31. In HepG2-H group, tumor showed higher microvessel density compared with HepG2 group (Fig. 3c). In addition, the lower expression of E-cadherin was also found in HepG2-H cells. Metformin increased the expression of E-cadherin, and reduced tumor microvessel density in HepG2 and HepG2-H tumors (Fig. 3c).

### Metfromin did not cause apparent toxicity in nude mice bearing with tumor

**Fig. 4:**
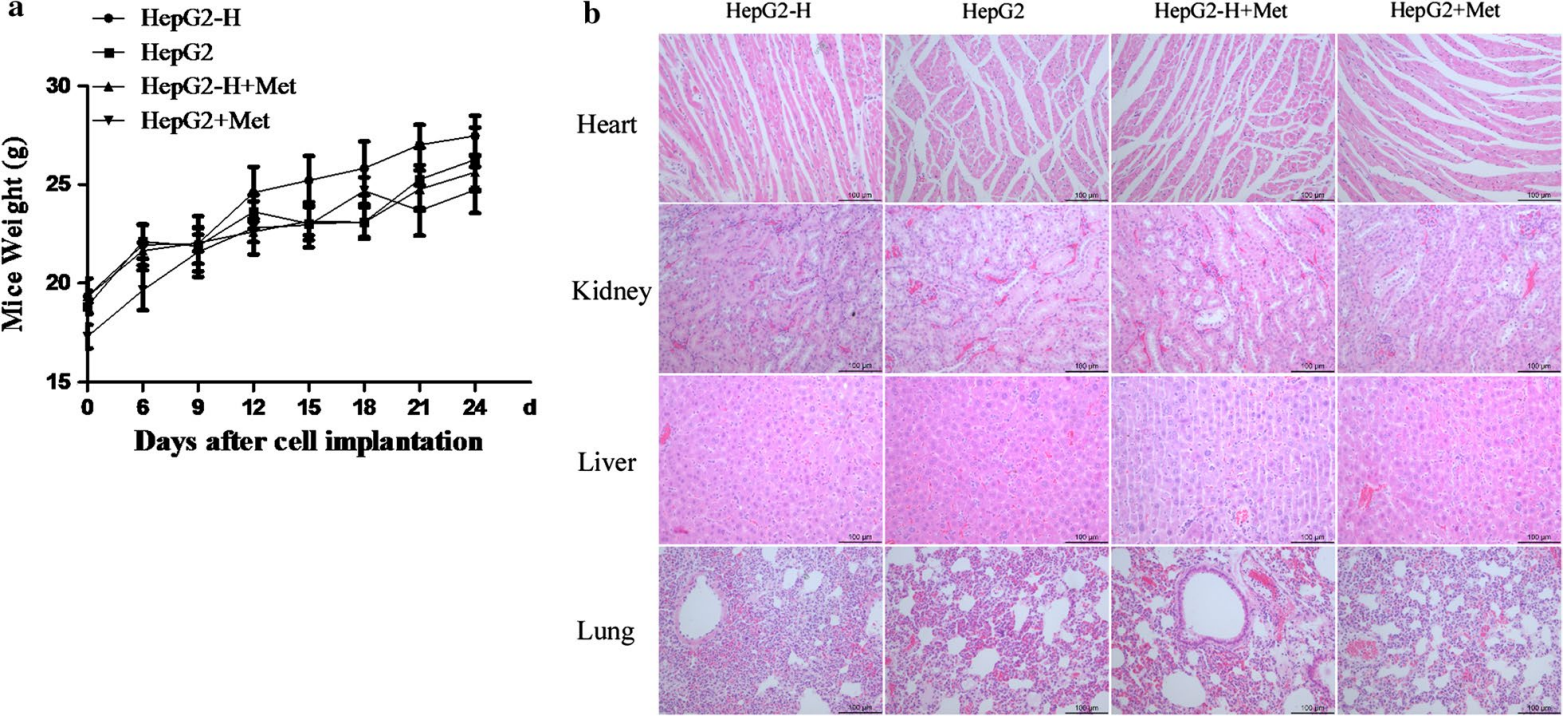
Toxicity of metformin on nude mice bearing with tumor. **a** Mice weight was measured with a scale every 3 days. **b** Heart, lung, liver and kidney sections were stained with haematoxylin and eosin (HE). Representative images were shown (200×). *ns* no significance

Metfromin had no significant effect on the mice body weight (Fig. 4a). Meanwhile, there were no apparent changes in liver, heart, kidney and lung in the mice (Fig. 4b).

## Discussion

Diabetes is now considered an independent risk factor for HCC [24]. Even more, diabetes may be a promoter for the progression of HCC after insufficient RFA. Metformin is the first-line drug for T2DM patients. Hence, in the present study, we demonstrated that metformin significantly inhibited growth of HCC cells after insufficient RFA in vitro and vivo. Our study may highlight the potential application of metformin in HCC after RFA, especially in patients with diabetes.

Metformin exert a direct effect on cancer cells partially through the activation of AMPK. Furthermore, the mTOR/AMPK pathway is the main mechanism of action of metformin [25]. The mTOR pathway plays a major role in tumor initiation and progression because of its involvement in multiple carcinogenic events such as cell growth, proliferation, survival and metabolism as well as protein biosynthesis and angiogenesis by a hypoxia-inducible factor 1 and a VEGF (vascular endothelial growth factor) dependent manner [26]. mTOR can also be phosphorylated by phosphorylated p-Akt-serine (S)473 to form p-mTOR-S2448, which positively regulates protein translation through the phosphorylation of its substrates, protein S6 kinase (p70S6K), and eukaryotic initiation factor 4E-binding protein 1 (4EBP1) [27]. Our previous study showed that HCC cells after insufficient RFA exhibited higher expression of p-Akt, which may play a key role in the EMT, and sorafenib suppressed the activity of p-Akt [5, 23]. In the present study, metformin up-regulated the expression of p-AMPK and PTEN, down-regulated the expression of p-Akt in HCC cells after insufficient RFA, and further down-regulated the increased expression of PCNA and VEGF in HCC cells after insufficient RFA. Therefore, metformin may play the part in the process through AMPK/PTEN/Akt signaling pathway.

The extensive use of metformin with nearly 120 million prescriptions worldwide each year is due to its favourable benefit-risk profile [28]. The glucose-lowering effect induced by metformin is clinically associated with a superior safety profile related to less cardiac mortality and rare cases of lactic acidosis at therapeutic doses. Moreover, compared to other anti-diabetic agents, metformin does not induce hypoglycaemia or weight gain [29]. In our study, metformin showed no cytotoxic effect on mice and did not cause weight loss. Likewise, if metformin could be applied to prevent HCC recurrence and metastasis after RFA, its cost-effectiveness is superior to other targeted therapy or chemotherapy.

Many clinical trials demonstrated that metformin could reduce the incidence and recurrence of HCC. Donadon et al. demonstrated that metformin reduced the HCC risk in type 2 diabetic patients [30, 31]. Hassan et al. [32] also suggested that metformin reduced the incidence of HCC in type 2 diabetic patients in a hospital-based case–control study in the United States. Lee et al. [33] identified benefits of metformin for HCC prevention compared to other anti-diabetics, with a reduced risk of other tumors, pancreatic and colorectal cancer as well. Lai [34] showed metformin may reduce the risk of development HCC among diabetic patients. Chan et al. reported that the use of metformin significantly reduces the risk of HCC recurrence and improves the overall outcome of patients after liver resection if patients survive the initial 2 years [13]. Chen et al. also showed that metformin users among diabetic patients with HCC undergoing RFA had a favorable overall survival compared with patients without metformin treatment [35]. In our study, we showed that HCC cells after insufficient RFA exhibited larger tumor volume compared with HCC cells after control treatment, and metformin inhibited the growth of HCC cells and eliminated the difference of growth in HepG2-H and HepG2 cells. So metformin might be used to prevent the progression of HCC cells after insufficient RFA. However, the researches about the strategy to prevent the progression of HCC after RFA are limited. In the present study, we only provided the preliminary basis for the application of metformin to prevent the progression of HCC cells after RFA, and more mechanisms involved in the progression and clinical trials should be investigated in the future.

## Conclusions

The study demonstrated that metformin inhibited the growth of HCC cells after insufficient RFA, and may be used to prevent the progression of HCC after RFA.

## Additional files

**Additional file 1: Figure S1.** Metformin suppressed the insufficient RFA-induced proliferation, migration and invasion of SMMC7721 cells. SMMC7721 cells were treated with insufficient RFA (47 °C 5, 10, 15, 20 and 25 min) gradually. Residual SMMC7721 (named as SMMC7721-H) cells were collected and used for the next experiments. (A) The effect of metformin on proliferation rate of SMMC7721 and SMMC7721-H cells was evaluated by MTT assay. Error bars represent the SEM of data obtained in five independent experiments. (B) Colony formation ability of SMMC7721 and SMMC7721-H cells after the treatment of metformin was assessed. (C) The effect of metfromin on migration and invasion of SMMC7721 and SMMC7721-H cells were shown. Error bars represent the SEM of data obtained in three independent experiments. ns, no significance.

**Additional file 2: Figure S2.** Metformin may regulate SMMC7721 cells proliferation, migration and invasion after insufficient RFA by promoting AMPK/PTEN/Akt pathway. Metformin was used to treat SMMC7721 cells, and western blot was used to determined the expression of p-AMPK, PTEN, p-Akt, E-cadherin, PCNA and VEGF.

## Abbreviations

RFA: radiofrequency ablation
HCC: hepatocellular carcinoma
DMEM: Dulbecco’s modified Eagle medium
FBS: fetal bovine serum
HRP: horseradish peroxidase
OD: optical density
VEGF: vascular endothelial growth factor
T2DM: Type 2 Diabetes Mellitus
