# Supplementary figures and images for: Metformin exhibits the anti-proliferation and anti-invasion effects in hepatocellular carcinoma cells after insufficient radiofrequency ablation
Source: Cancer Cell Int. 2017 Apr 24;17:48. doi: 10.1186/s12935-017-0418-6 (PMC5404300; doi:10.1186/s12935-017-0418-6)

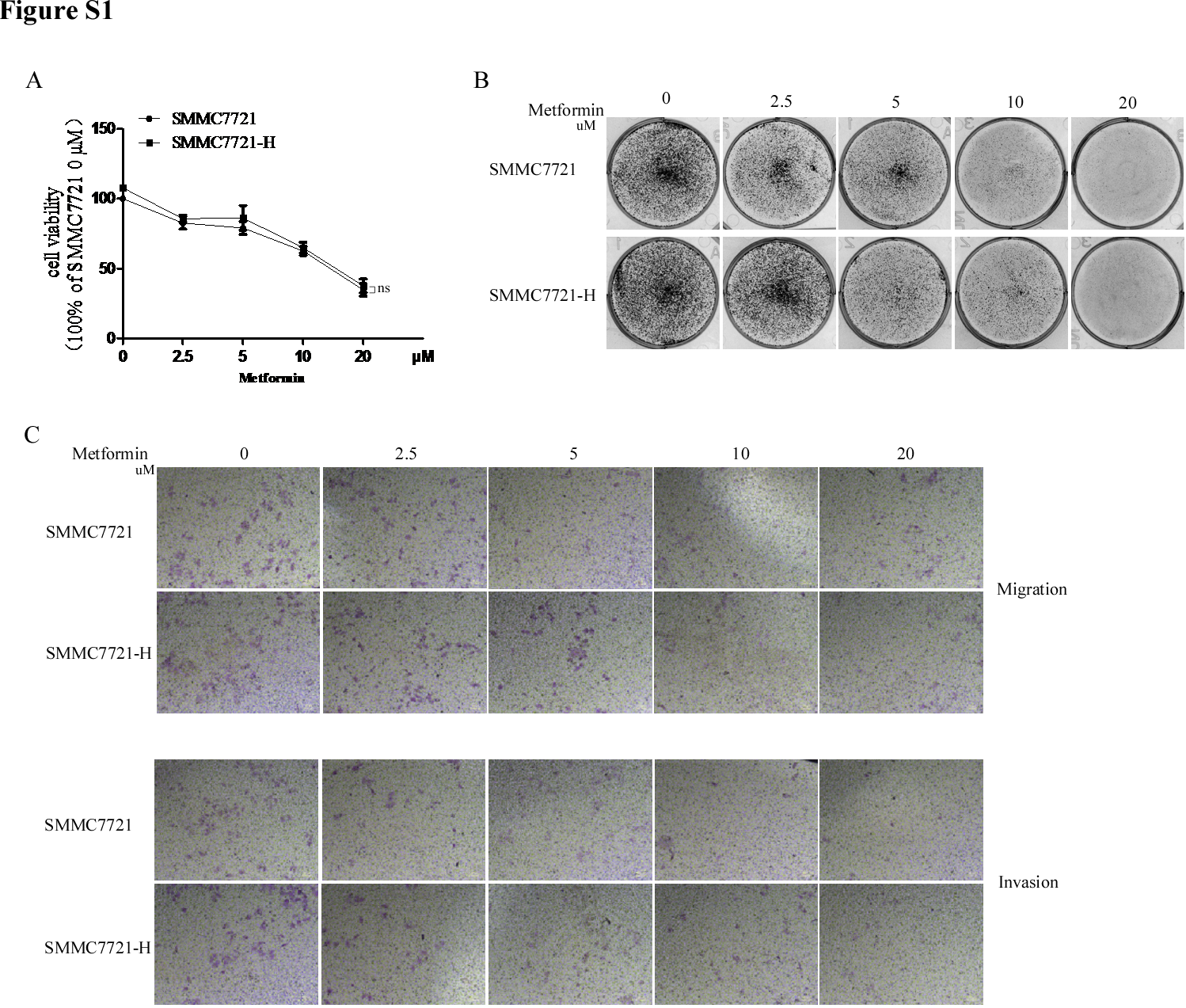

Supplement: Supplementary file 1 — Additional file 1: Figure S1. Metformin suppressed the insufficient RFA-induced proliferation, migration and invasion of SMMC7721 cells. SMMC7721 cells were treated with insufficient RFA (47 °C 5, 10, 15, 20 and 25 min) gradually. Residual SMMC7721 (named as SMMC7721-H) cells were collected and used for the next experiments. (A) The effect of metformin on proliferation rate of SMMC7721 and SMMC7721-H cells was evaluated by MTT assay. Error bars represent the SEM of data obtained in five independent experiments. (B) Colony formation ability of SMMC7721 and SMMC7721-H cells after the treatment of metformin was assessed. (C) The effect of metfromin on migration and invasion of SMMC7721 and SMMC7721-H cells were shown. Error bars represent the SEM of data obtained in three independent experiments. ns, no significance. [file 12935_2017_418_MOESM1_ESM.tif]

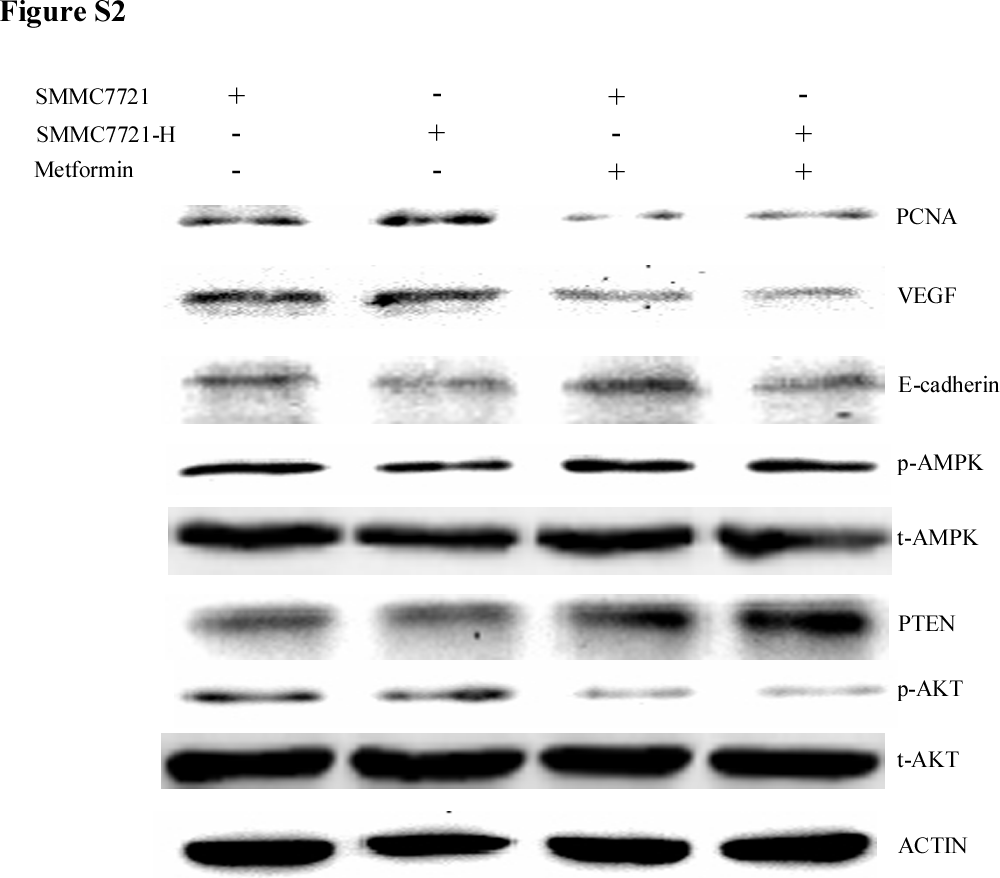

Supplement: Supplementary file 2 — Additional file 2: Figure S2. Metformin may regulate SMMC7721 cells proliferation, migration and invasion after insufficient RFA by promoting AMPK/PTEN/Akt pathway. Metformin was used to treat SMMC7721 cells, and western blot was used to determined the expression of p-AMPK, PTEN, p-Akt, E-cadherin, PCNA and VEGF. [file 12935_2017_418_MOESM2_ESM.tif]
